# Bias in trials comparing paired continuous tests can cause researchers to choose the wrong screening modality

**DOI:** 10.1186/1471-2288-9-4

**Published:** 2009-01-20

**Authors:** Deborah H Glueck, Molly M Lamb, Colin I O'Donnell, Brandy M Ringham, John T Brinton, Keith E Muller, John M Lewin, Todd A Alonzo, Etta D Pisano

**Affiliations:** 1 Department of Biostatistics, Colorado School of Public Health, University of Colorado, Denver, Aurora, CO, USA; 2 Department of Epidemiology, Colorado School of Public Health, University of Colorado, Denver, Aurora, CO, USA; 3 Division of Biostatistics, Department of Epidemiology and Health Policy Research, University of Florida, Gainesville, FL, USA; 4 Diversified Radiology of Colorado, Denver, CO, USA; 5 Department of Preventive Medicine, University of Southern California, Arcadia, CA, USA; 6 Departments of Radiology and Biomedical Engineering, the Biomedical Research Imaging Center and the Lineberger Comprehensive Cancer Center, University of North Carolina at Chapel Hill, Chapel Hill, NC, USA

## Abstract

**Background:**

To compare the diagnostic accuracy of two continuous screening tests, a common approach is to test the difference between the areas under the receiver operating characteristic (ROC) curves. After study participants are screened with both screening tests, the disease status is determined as accurately as possible, either by an invasive, sensitive and specific secondary test, or by a less invasive, but less sensitive approach. For most participants, disease status is approximated through the less sensitive approach. The invasive test must be limited to the fraction of the participants whose results on either or both screening tests exceed a threshold of suspicion, or who develop signs and symptoms of the disease after the initial screening tests.

The limitations of this study design lead to a bias in the ROC curves we call *paired screening trial bias*. This bias reflects the synergistic effects of inappropriate reference standard bias, differential verification bias, and partial verification bias. The absence of a gold reference standard leads to inappropriate reference standard bias. When different reference standards are used to ascertain disease status, it creates differential verification bias. When only suspicious screening test scores trigger a sensitive and specific secondary test, the result is a form of partial verification bias.

**Methods:**

For paired screening tests with bivariate normally distributed scores, we give formulae and programs to quantify the effect of *paired screening trial bias *on a paired comparison of area under the curves. We fix the prevalence of disease, and the chance a diseased subject manifests signs and symptoms. We derive the formulas for true sensitivity and specificity, and those for the sensitivity and specificity observed by the study investigator.

**Results:**

The observed area under the ROC curves is quite different from the true area under the ROC curves. The typical direction of the bias is a strong inflation in sensitivity, paired with a concomitant slight deflation of specificity.

**Conclusion:**

In paired trials of screening tests, when area under the ROC curve is used as the metric, bias may lead researchers to make the wrong decision as to which screening test is better.

## Background

Paired trials designed to compare the diagnostic accuracy of screening tests using area under the receiver operating characteristic (ROC) curve may fall victim to a strong bias that renders the conclusions of the trial incorrect. In English, "bias" often has a pejorative connotation, implying that those who conduct the study prefer one scientific conclusion, rather than another. We use the term "bias" in the epidemiological and statistical sense, as the difference between the results obtained in a study, and the true results.

The bias occurs because limitations in the trial design may differentially affect the area under the ROC curve for each screening test. Many competing statistical approaches have been suggested for comparing the diagnostic accuracy of two continuous tests. We consider area under the ROC curve, because it continues to be used as the standard in prominent medical journals [1-3].

A common design for the comparison of two continuous screening tests is to evaluate participants with both screening tests. The disease status is then determined by either an invasive secondary test, or by a less invasive, but less sensitive approach. Ethically and practically, the invasive secondary test must be reserved only for those participants who have a suspicious result on one or both screening tests, or for those who have signs and symptoms of disease. For those who have a normal result on both screening tests, a less sensitive process is used to approximate the disease status. As the *true *disease status is not known correctly for all participants, the *observed *disease status is used for calculations of diagnostic accuracy.

For potentially lethal diseases like cancer, where the invasive test is biopsy, this design is the best possible available design. The imperfections of the study design occur because the disease is difficult to diagnose since it is clinically occult, and the study designers must keep the risk of potential harm to subjects as low as possible.

The limitations of this design leads to a previously undescribed bias we call *paired screening trial bias*. This bias results from the synergistic effects of inappropriate reference standard bias, differential verification bias, and partial verification bias [4]. Here, verification is used to describe the process of ascertaining the disease status. In classical partial verification bias, only some participants undergo determination of disease status. A variant of partial verification bias is extreme verification bias, when only strongly abnormal results on one of the screening tests lead to secondary testing [5]. In the paired screening trial design we discuss here, an effect similar to partial verification bias operates. A disease status is assigned for all participants, but determined with great sensitivity and specificity only for those with strongly abnormal results on an initial screening test. Because different methods are used to ascertain disease status, depending on the results of the initial screening tests, the trial is subject to differential verification bias. Finally, paired screening trials often yield fewer *observed *than *true *cases of disease. Some cases of disease are missed because the ascertainment of disease status is not perfect. Thus, the trial is subject to inappropriate reference standard bias. All three of these biases interact to inflate the sensitivity and to slightly deflate the specificity, in potentially differential amounts for each screening test.

When differentially biased estimates of sensitivity and specificity are used to construct receiver operating characteristic (ROC) curves for the two screening tests, the resulting areas under the ROC curves are also incorrect. Therefore, when tests are used to compare the areas under the ROC curves, the conclusions drawn regarding the relative diagnostic accuracy of the two tests may be wrong. This potential pitfall has strong clinical implications because a paired comparison of areas under ROC curves is one of the most common tests used to compare screening modalities. Thus, *paired screening trial bias *may have a large impact on the design and interpretation of screening trials. We provide formulas to quantify the bias. We describe the conditions that cause incorrect scientific conclusions as to which screening modality is better. We also demonstrate that paired screening trial bias may not affect the scientific conclusion, and explain when the scientific conclusion is likely to be correct.

## Methods

### Study design

We consider a hypothetical trial in which each subject receives two screening tests at the same time in a paired design. In the trial, the disease status is determined either by a secondary, sensitive and specific but invasive test, like biopsy, or approximated by a less sensitive process, like follow-up for a certain time period. The diagnostic accuracy of the two screening tests is to be compared using a paired comparison for the difference in area under the ROC curve for each screening test.

There are two possible viewpoints for the trial. One is omniscient, in which the *true *disease status is known for each subject. The other is the viewpoint of the study investigator, who observes the disease status with error due to the limitations of the trial design. Because we use a mathematical model, we can derive the probability of all outcomes from each point of view.

**Figure 1:**
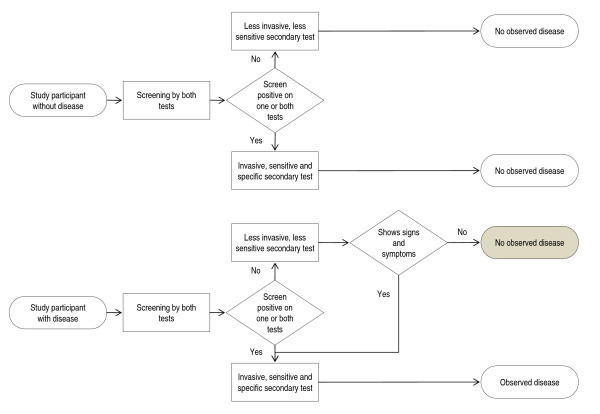
**Paired screening trial flowchart**. Trial design, and observed and true outcomes for a paired screening trial of two continuous tests, with two possible secondary tests used determine disease status. Cases of disease which escape detection during the study appear in the shaded oval. The study investigator will miss cases of disease when 1) no signs or symptoms are observed; 2) the less sensitive secondary test is used; and 3) the participant has disease. One or both screening tests may cause a participant without disease to be recalled. By assumption, the invasive, sensitive and specific secondary test will never diagnose disease when there is in fact no disease. Thus, the study investigator never declares a participant to have disease when in fact they do not.

A flow chart of the hypothetical study is shown in Figure 1. Disease is observed by the study investigator in one of four ways. 1) A patient has an abnormal result on screening Test 1 only and then has an abnormal secondary test, leading to the diagnosis of disease. 2) A patient has an abnormal result on screening Test 2 only and then has an abnormal secondary test, leading to the diagnosis of disease. 3) A patient has abnormal results on both screening Test 1 and screening Test 2 and then has an abnormal secondary test, leading to the diagnosis of disease. 4) A patient has normal results on both screening Test 1 and screening Test 2, and thus no secondary test, but later presents with signs and symptoms, which lead to an abnormal secondary test, and the subsequent diagnosis of disease.

In this analysis, we will refer to the disease status observed in the study as the *observed disease status *and the true disease status as the *true disease status*, with *observed *and *true *as shorthand, respectively. We quantify bias by examining the difference between the ROC curves drawn using the *observed *disease status, and those drawn using the *true *disease status.

### Model, Assumptions and Definitions

We model the potential errors due to *paired screening trial bias *for this hypothetical trial. A series of assumptions allow us to examine the potential impact of *paired screening trial bias *in a situation with no experimental noise.

First, we assume that the results of screening Test 1 and screening Test 2 have a bivariate normal distribution for the participants with disease, and a potentially different bivariate normal distribution for the participants without disease. While normally distributed data is not typically observed in studies, the assumption of normality underlies the popular ROC analysis technique of Metz *et al*. [6].

Suppose that the variance, *σ*^2^, is the same for both distributions. The equal variance assumption prevents the true ROC curves from crossing. Let *μ*_*C*1 _and *μ*_*C*2 _be the mean scores for participants with disease given by screening Test 1 and screening Test 2, and *μ*_*N*1 _and *μ*_*N*2 _be the mean scores for participants without disease given by screening Test 1 and screening Test 2, respectively. Suppose *ρ*_*C *_is the correlation between the two test scores for participants with disease, and *ρ*_*N *_is the correlation between the two test scores for participants without disease. Scores for different participants were assumed to be independent.

We assume that a high score on a screening test results in an increased level of suspicion. We define *x *to be the cutpoint for each 2 × 2 table that defines the ROC curve. Scores above *x *are declared positive on each test, while scores below *x *are declared negative. We assume that the invasive, yet sensitive and specific secondary test never misses disease when disease is present. Likewise, if a subject has no disease, the invasive, yet sensitive and specific secondary test always correctly indicates that the subject is disease free. We also assume that all test scores above a pre-specified threshold lead to the invasive, yet sensitive and specific secondary test. *θ *is the value of the test score above which participants must have the invasive, yet sensitive and specific secondary test. We will call *θ *the threshold for recall. All participants who do not undergo the invasive, yet sensitive and specific secondary test have a less sensitive, but less invasive secondary test, such as follow-up.

For convenience in the derivation, we use the same value of the threshold for recall, *θ*, for both screening tests. Because ROC analysis is invariant to translation, choosing the same values of *θ *for each screening test, and then shifting the means of the screening test scores has the same mathematical result as choosing different values of *θ *for each screening test.

During the follow-up period, some participants will experience signs and symptoms of disease. We assume that only participants with disease will experience signs and symptoms of disease. Participants who experience signs and symptoms of disease are then given the invasive, yet sensitive and specific secondary test, which we have previously assumed is infallible. For participants with signs and symptoms, the study investigator always observes the correct outcome. The study investigator incorrectly specifies that a participant has no disease when all three of the following conditions are met: 1) the participant has disease, 2) the participant scores below *θ *on both screening tests, avoiding the invasive, yet sensitive and specific secondary test, and 3) never experiences signs or symptoms during the follow-up period.

The prevalence of disease in the population is *r*. The proportion of participants with disease who experience signs and symptoms within the study follow-up period, but not at study entry, is *ψ*. We write Φ(*x*) to indicate the cumulative distribution function of a normal distribution with mean 0 and standard deviation 1, evaluated at the point *x*, and Φ(*x*, *y*, *ρ*) to indicate the cumulative distribution function of a bivariate normal distribution with mean vector [0, 0], standard deviations both 1 and correlation *ρ*, evaluated at the points *x *and *y*. That is, if *X *and *Y *have a bivariate normal distribution, we write Φ(*x*, *y*, *ρ*) to indicate Pr (*X *≤ *x *and *Y *≤ *y*|$\sigma_{X}^{2}$, $\sigma_{Y}^{2}$ = 1, *ρ*)

The data are paired, so there are two observed test scores for each subject. By assumption, the two scores are correlated. Each test score could fall above or below *θ*, the threshold value for referral to the invasive, yet sensitive and specific secondary test. Thus, for each value of *x*, we can describe a series of events cross-classified by the Test 1 score, the Test 2 score, the true disease status of the subject and the presence of signs or symptoms. We classify each event both as it truly occurs, and how it is observed by the study investigator. There are 22 possible situations when *x *<*θ *(Table 1), and 19 such situations when *x *> *θ *(Table 2).

**Table 1:** For *x *<*θ*, observed screening test results, and observed and true disease status.

Test 1 Score Inequality	Test 1 Result	Test 2 Score Inequality	Test 2 Result	Signs and Symptoms	Observed Disease Status	True Disease Status
*T*_1 _<*x*	-	*T*_2 _<*x*	-		-	-
*T*_1 _<*x*	-	*T*_2 _<*x*	-	No signs	-	+
*T*_1 _<*x*	-	*T*_2 _<*x*	-	Signs	+	+
*T*_1 _<*x*	-	*x *<*T*_2 _<*θ*	+		-	-
*T*_1 _<*x*	-	*x *<*T*_2 _<*θ*	+	No signs	-	+
*T*_1 _<*x*	-	*x *<*T*_2 _<*θ*	+	Signs	+	+
*T*_1 _<*x*	-	*θ *<*T*_2_	+		-	-
*T*_1 _<*x*	-	*θ *<*T*_2_	+		+	+
*x *<*T*_1 _<*θ*	+	*T*_2 _<*x*	-		-	-
*x *<*T*_1 _<*θ*	+	*T*_2 _<*x*	-	No signs	-	+
*x *<*T*_1 _<*θ*	+	*T*_2 _<*x*	-	Signs	+	+
*x *<*T*_1 _<*θ*	+	*x *<*T*_2 _<*θ*	+		-	-
*x *<*T*_1 _<*θ*	+	*x *<*T*_2 _<*θ*	+	No signs	-	+
*x *<*T*_1 _<*θ*	+	*x *<*T*_2 _<*θ*	+	Signs	+	+
*x *<*T*_1 _<*θ*	+	*θ *<*T*_2_	+		-	-
*x *<*T*_1 _<*θ*	+	*θ *<*T*_2_	+		+	+
*θ *<*T*_1_	+	*T*_2 _<*x*	-		-	-
*θ *<*T*_1_	+	*T*_2 _<*x*	-		+	+
*θ *<*T*_1_	+	*x *<*T*_2 _<*θ*	+		-	-
*θ *<*T*_1_	+	*x *<*T*_2 _<*θ*	+		+	+
*θ *<*T*_1_	+	*θ *<*T*_2_	+		-	-
*θ *<*T*_1_	+	*θ *<*T*_2_	+		+	+

The situations listed in this table are those where *x *<*θ*, i.e. that the test score is less than the threshold that leads to referral to the invasive, yet sensitive and specific secondary test. The signs and symptoms column is left empty in those cases where the observed disease status does not depend on the development of signs or symptoms.

**Table 2:** For *x *> *θ*, observed screening test results, and observed and true disease status.

Test 1 Score Inequality	Test 1 Result	Test 2 Score Inequality	Test 2 Result	Signs and Symptoms	Observed Disease Status	True Disease Status
*T*_1 _<*θ*	-	*T*_2 _<*θ*	-		-	-
*T*_1 _<*θ*	-	*T*_2 _<*θ*	-	No signs	-	+
*T*_1 _<*θ*	-	*T*_2 _<*θ*	-	Signs	+	+
*T*_1 _<*θ*	-	*θ *<*T*_2 _<*x*	-		-	-
*T*_1 _<*θ*	-	*θ *<*T*_2 _<*x*	-		+	+
*T*_1 _<*θ*	-	*x *<*T*_2_	+		-	-
*T*_1 _<*θ*	-	*x *<*T*_2_	+		+	+
*θ *<*T*_1 _<*x*	-	*T*_2 _<*θ*	-		-	-
*θ *<*T*_1 _<*x*	-	*T*_2 _<*θ*	-		+	+
*θ *<*T*_1 _<*x*	-	*θ *<*T*_2 _<*x*	-		-	-
*θ *<*T*_1 _<*x*	-	*θ *<*T*_2 _<*x*	-		+	+
*θ *<*T*_1 _<*x*	-	*x *<*T*_2_	+		-	-
*θ *<*T*_1 _<*x*	-	*x *<*T*_2_	+		+	+
*x *<*T*_1_	+	*T*_2 _<*θ*	-		-	-
*x *<*T*_1_	+	*T*_2 _<*θ*	-		+	+
*x *<*T*_1_	+	*θ *<*T*_2 _<*x*	-		-	-
*x *<*T*_1_	+	*θ *<*T*_2 _<*x*	-		+	+
*x *<*T*_1_	+	*x *<*T*_2_	+		-	-
*x *<*T*_1_	+	*x *<*T*_2_	+		+	+

The situations listed in this table are those where *x *> *θ*, i.e. that the test score is greater than the threshold that leads to referral to the invasive, yet sensitive and specific secondary test. The signs and symptoms column is left empty in those cases where the observed disease status does not depend on the development of signs or symptoms

For each screening test and each value of the test cutpoint *x*, we can define a table that cross classifies the response of the test (positive or negative), and the truth (the presence or absence of disease). The cell and marginal probabilities for this cross-classification are shown in Tables 3 and 4. We obtain the probabilities in two steps. First, we use our model, assumption and definitions to assign probabilities to each situation shown in Tables 1 and 2. Then, using the disease status and screening test results to classify the events in Tables 1 and 2 into the appropriate four groups, we sum the appropriate event probabilities to obtain the cell and marginal probabilities shown in Tables 3 and 4. For example, in Table 3, the screening Test 1 +, true disease + cell has the probability formed by summing all entries in Table 1 where screening Test 1 is + and the subject has disease.

**Table 3:** True disease status and Test 1 results.

		True Disease Status	
		+	-
Test 1	+	*r *[1 - Φ (*x *- *μ*_*C*1_)]	(1 - *r*) [1 - Φ (*x *- *μ*_*N*1_)]
	-	*r *Φ (*x *- *μ*_*C*1_)]	(1 - *r*) Φ (*x *- *μ*_*N*1_)

Test 1 is + if the score on Test 1 is greater than *x*.

**Table 4:** True disease status and Test 2 results.

		True Disease Status	
		+	-
Test 2	+	*r *[1 - Φ (*x *- *μ*_*C*2_)]	(1 - *r*) [1 - Φ (*x *- *μ*_*N*2_)]
	-	*r *Φ (*x *- *μ*_*C*2_)]	(1 - *r*)Φ (*x *- *μ*_*N*2_)

Test 2 is + if the score on Test 1 is greater than *x*.

We then calculate the *true *sensitivity for each test as the number of true positives identified by that screening test divided by the total number of true cases. The *true *specificity for each test is the number of true negatives correctly identified as negative by that screening test divided by the total number of true non-cases. The *true *ROC curve is generated by plotting the *true *sensitivity on the vertical axis versus one minus the *true *specificity on the horizontal axis.

We use a similar technique to calculate the *observed *sensitivity and *observed *specificity. In order to generate the *observed *ROC curves, for each test and each value of the cutpoint *x*, we define a table that cross classifies the response of the test (positive or negative), and the *observed *disease status (the presence or absence of *observed *disease). The cell and marginal probabilities for this cross-classification are shown in Tables 5 and 6. We then calculate the *observed *sensitivity and the *observed *specificity. The *observed *sensitivity is the fraction of participants *observed *to have disease who have a positive screening test result. The *observed *specificity is the fraction of participants who apparently have no disease who have a negative screening test result. Some participants actually may have disease, but the disease is not detected in the trial.

**Table 5:** Observed disease status and Test 1 results.

Test 1	Observed Disease Status	Probability
+	+	$\{\begin{array}{ll} r[1-\Phi (x-\mu_{C1})]+r(1-\psi )\times & \\ {}[\Phi (x-\mu_{C1},\theta -\mu_{C2},\rho_{C})-\Phi (\theta -\mu_{C1},\theta -\mu_{C2},\rho_{C})] & x<\theta \\ r[1-\Phi (x-\mu_{C1})] & x\geq \theta \end{array}$
+	-	$\{\begin{array}{ll} (1-r)[1-\Phi (x-\mu_{N1})]-r(1-\psi )\times & \\ {}[\Phi (x-\mu_{C1},\theta -\mu_{C2},\rho_{C})-\Phi (\theta -\mu_{C1},\theta -\mu_{C2},\rho_{C})] & x<\theta \\ \left(1-r)[1-\Phi (x-\mu_{N1})\right] & x\geq \theta \end{array}$
-	+	$\{\begin{array}{ll} r\Phi (x-\mu_{C1})- & \\ r(1-\psi )\Phi (x-\mu_{C1},\theta -\mu_{C2},\rho_{C}) & x<\theta \\ r\Phi (x-\mu_{C1})- & \\ r(1-\psi )\Phi (\theta -\mu_{C1},\theta -\mu_{C2},\rho_{C}) & x\geq \theta \end{array}$
-	-	$\{\begin{array}{ll} (1-r)\Phi (x-\mu_{N1})+ & \\ r(1-\psi )\Phi (x-\mu_{C1},\theta -\mu_{C2},\rho_{C}) & x<\theta \\ (1-r)\Phi (x-\mu_{N1})+ & \\ r(1-\psi )\Phi (\theta -\mu_{C1},\theta -\mu_{C2},\rho_{C}) & x\geq \theta \end{array}$

Test 1 is + if the score on Test 1 is greater than *x*.

**Table 6:** Observed disease status and Test 2 results.

Test 2	Observed Disease Status	Probability
+	+	$\{\begin{array}{ll} r[1-\Phi (x-\mu_{C2})]+r(1-\psi )\times & \\ {}[\Phi (\theta -\mu_{C1},x-\mu_{C2},\rho_{C})-\Phi (\theta -\mu_{C1},\theta -\mu_{C2},\rho_{C})] & x<\theta \\ r[1-\Phi (x-\mu_{C2})] & x\geq \theta \end{array}$
+	-	$\{\begin{array}{ll} (1-r)[1-\Phi (x-\mu_{N2})]-r(1-\psi )\times & \\ {}[\Phi (\theta -\mu_{C1},x-\mu_{C2},\rho_{C})-\Phi (\theta -\mu_{C1},\theta -\mu_{C2},\rho_{C})] & x<\theta \\ \left(1-r)[1-\Phi (x-\mu_{N2})\right] & x\geq \theta \end{array}$
-	+	$\{\begin{array}{ll} r\Phi (x-\mu_{C2})- & \\ r(1-\psi )\Phi (\theta -\mu_{C1},x-\mu_{C2},\rho_{C}) & x<\theta \\ r\Phi (x-\mu_{C2})- & \\ r(1-\psi )\Phi (\theta -\mu_{C1},\theta -\mu_{C2},\rho_{C}) & x\geq \theta \end{array}$
-	-	$\{\begin{array}{ll} (1-r)\Phi (x-\mu_{N2})+ & \\ r(1-\psi )\Phi (\theta -\mu_{C1},x-\mu_{C2},\rho_{C}) & x<\theta \\ (1-r)\Phi (x-\mu_{N2})+ & \\ r(1-\psi )\Phi (\theta -\mu_{C1},\theta -\mu_{C2},\rho_{C}) & x\geq \theta \end{array}$

Test 2 is + if the score on Test 2 is greater than *x*.

The *observed *ROC curve is generated by plotting the *observed *sensitivity on the vertical axis versus one minus the *observed *specificity on the horizontal axis. Simpson's rule numerical integration methods [[7], p. 608] with accuracy of 0.001 are used to calculate the area under the ROC curve (AUC) for each screening test.

We calculate the theoretically correct ROC curves and AUCs (ignoring the error of integration), using our mathematical derivations. In a real trial, the study investigator would use a hypothesis test and a p-value to compare the difference in AUCs. Depending on the sample size chosen for the trial, the precision of the estimates and the accuracy of the decision may change.

To illustrate the effect of the bias, we present the theoretical results. To illustrate the effect of sample size on the precision of the estimates, we conduct a simulation. For the simulation, we suppose that the study investigator decided to test the null hypothesis of no difference between the areas under the ROC curves, using a non-parametric AUC test for paired data [8], and fixing the Type I error rate at 0.05. To ensure adequate power, for a fixed set of parameters, we set the sample size so that 90% of the time, if the true state of disease were known, the null hypothesis would be rejected. For that fixed set of parameters and sample size, we simulate 10,000 sets of data. For both the *true *state of disease, and the *observed *state of disease, we record the magnitude of the differences in AUCs, and the decision whether to reject the null. The proportion of rejections for the *true *and *observed *data is estimated by the number of rejections, divided by 10,000. Ten thousand is chosen so the maximum half width for the confidence interval for the proportion rejected is no more than 0.01.

## Results

Our derivations demonstrate that the *observed *ROC curve differs from the *true *ROC curve, with the amount of bias depending on the correlation between the screening tests for participants with disease, *ρ*_*C*_, the rate of signs and symptoms, *ψ*, and the threshold for recall, *θ*. In some cases, the bias equally affects the observed ROC curve for both screening tests, and the scientific conclusion is the same as it would have been had the true disease state been observed. In other cases, the bias causes a change in the direction of the scientific conclusion. The scientific conclusion only changes direction when for one screening test, for participants with disease, a higher proportion of the scores lead to recall than for the other screening test. Thus, for that screening test, a larger percent of participants with true disease go on to have their disease status correctly ascertained, and observed in the study, than for the other screening test.

**Figure 2:**
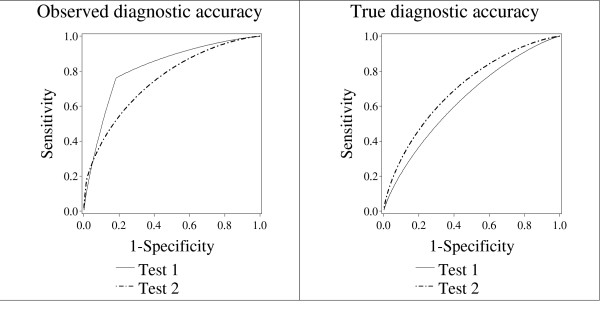
**True and observed ROC curves for a hypothetical example where bias changes the scientific conclusion**. The parameters for this example were chosen to illustrate a case where paired screening trial bias may cause an incorrect scientific conclusion. The incorrect conclusion occurs because for participants with disease, one screening test leads to a higher chance of recalls than the other screening test. The chance of recall for Test 2 for a participant who had disease was 34%, while for Test 1 it was 8%. For this example, we fixed the disease rate, *r *= 0.01; the chance that participants with disease would experience signs and symptoms within the year of follow-up, *ψ *= 0.1; the variance, *σ*^2 ^= 1. The means of the distributions of test results for cases for Test 1 and Test 2 were 2.1 and 1.1, respectively, and the means for non-cases for Test 1 and Test 2 were 1.6 and 0.35. The correlation between test scores for cases was fixed at 0.1, as was the correlation for non-cases. All test scores above 2.5 on either test, or participants who had signs or symptoms had an infallible secondary test to determine disease status. For participants with scores below 2.5 on both tests, a less sensitive method was used to approximate disease status.

**Figure 3:**
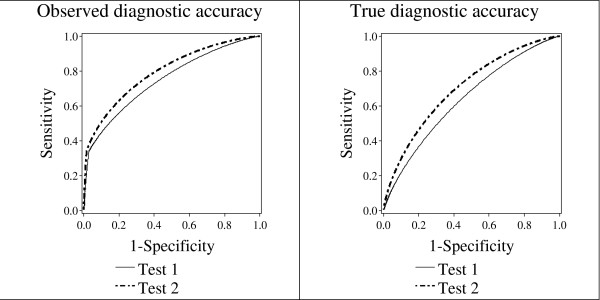
**True and observed ROC curves for a hypothetical example where bias did not change the scientific conclusion**. The parameters for this example were chosen to illustrate a case where paired screening trial bias did not change the direction of the difference in AUC, nor the scientific conclusion. The chance of recall for either screening test for a participant who had disease was 8%. For this example, we fixed the disease rate, *r *= 0.01; the chance that participants with disease would experience signs and symptoms within the year of follow-up, *ψ *= 0.1; the variance, *σ*^2 ^= 1. The means of the distributions of test results for cases for Test 1 and Test 2 were 1.1 and 1.1, respectively, and the means for non-cases for Test 1 and Test 2 were 1.6 and 0.35. The correlation between test scores for cases was fixed at 0.1, as was the correlation for non-cases. All test scores above 2.5 on either test, or participants who had signs or symptoms had an infallible secondary test to determine disease status. For participants with scores below 2.5 on both tests, a less sensitive method was used to approximate disease status.

Figure 2 and Figure 3 demonstrate the possible effects of bias on the scientific conclusions. In Figure 2, the study investigator will draw the wrong scientific conclusion. In Figure 3, the study investigator will draw the correct scientific conclusion, despite the presence of bias. For Figure 3, the participants with the highest 8% of both screening Test 1 and screening Test 2 scores will be recalled for the sensitive and specific secondary test. For Figure 2, the participants with the highest 34% of the screening test scores for Test 1 will be recalled, but only the highest 8% for Test 2. In general, the scientific conclusion is correct when both screening tests lead to a secondary test at the same rate. The scientific conclusion may be wrong when the chance of proceeding to the secondary test depends on which screening test produced a high score.

As shown in Figure 2 and Figure 3, the *observed *curves have inflection points, where the slope changes. There is no inflection point in the true ROC curves for either test, because the formulae that govern the sensitivity and specificity for the *true *curves are the same no matter what the ROC cutoff points are (see Tables 3 and 4). By contrast, as shown in Tables 5 and 6, the formulae for the *observed *ROC curves change depending on whether the cutpoint is above or below *θ*. This causes a change in slope for the *observed *ROC curve. The inflection point is more obvious for Test 2 than for Test 1. The inflection point for Test 1 occurs at specificity of about 0.80, and is obscured in Figure 2. In general, as *θ *increases relative to the mean of the test score distribution, the point of inflection occurs at higher values of specificity.

In Figure 2, the *true *ROC curve for screening Test 2 is higher than the *true *ROC curve for screening Test 1. Thus, screening Test 2 has better *true *diagnostic accuracy than screening Test 1. However the *observed *ROC curve for screening Test 1 is higher than the *observed *ROC curve for Test 2.

In Figure 2, bias in the *observed *ROC curves leads to a bias in the *observed *AUC for each test. Recall that in reality, screening Test 2 has better diagnostic accuracy than screening Test 1. The *true *AUC of screening Test 1 is 0.64, and the *true *AUC of screening Test 2 is 0.70. However, the *observed *AUC tells a different story. The *observed *AUC for screening Test 1 is 0.82, and the *observed *AUC for screening Test 2 is 0.75.

Since Test 2 truly has better diagnostic accuracy than Test 1, the *true *difference in AUC between screening Test 2 and Test 1 is positive (Test 2 *true *AUC – Test 1 *true *AUC = 0.70 - 0.64 = 0.06). However, in Figure 2, the *observed *difference in AUC between Test 2 and Test 1 is negative (Test 2 observed AUC – Test 1 observed AUC = 0.75 – 0.82 = -0.07). If the study investigator were to observe these exact theoretical results, the study investigator would conclude that screening Test 1 has better diagnostic accuracy than Test 2, when in fact the opposite is true.

Study investigators never observe the true state of nature. They observe data, and make estimates, the precision of which depends on the sample size. They decide which screening test is better using hypothesis tests. To see which conclusion the hypothesis tests would suggest, both for the *true *and *observed *disease status, we conducted a simulation. For the parameters of Figure 2, for a Type 1 error rate of 0.05, if the true disease status were known, a non-parametric test [8] would have 90% power with 33,000 participants. With the true disease status known, we would reject the null roughly 90% of the time. The remaining 10% of the time, we would conclude no difference in AUC between Test 1 and Test 2. If the true disease status were known, every time we rejected the null, we would conclude correctly that Test 2 is better than Test 1.

If we conduct the same simulation experiment from the point of view of the study investigator, for the experimental situation of Figure 2, we see only the *observed *state of disease. In that case, the study investigator will reject the null hypothesis only 71% of the time. The remaining 29% of the time, the study investigator will conclude that there is no difference in AUC between Test 1 and Test 2. The lower power is due to more variance in the *observed *data, compared to the *true *data. When the study investigator rejects the null, every time, she concludes incorrectly that Test 1 is better than Test 2.

The incorrect conclusion in Figure 2 is the result of a cascade of errors. The *observed *sensitivity for Test 1 is inflated more than the *observed *sensitivity for Test 2. The increase in *observed *sensitivity makes the *observed *ROC curve higher for Test 1 than for Test 2. A higher *observed *ROC curve means a higher *observed *AUC for Test 1 than for Test 2.

**Figure 4:**
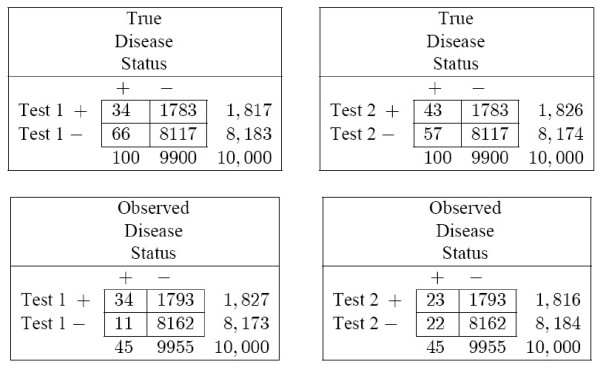
**For the hypothetical example of Figure 2, true and observed 2 × 2 tables**. Numbers were rounded to the nearest whole number. All tables were calculated at specificity of about 0.82. This point was chosen because the maximum difference between the observed and true sensitivity for Test 1 occurs at this point. For Test 1, the true sensitivity is 0.34, withobserved sensitivity at 0.76. For Test 2, the true sensitivity is 0.43, withobserved sensitivity at 0.51. Each one of the four tables uses a slightly different ROC cutpoint. For the observed table, Test 1 is positive if it exceeds 2.511; for the true table, Test 1 is positive if it exceeds 2.515. For the observed table, Test 2 is positive if it exceeds 1.269; for the true table, Test 2 is positive if it exceeds 1.265. The tables have different ROC cutpoints because they were chosen to have the same specificity, not the same cutpoint. For this hypothetical example, the disease rate, *r *= 0.01; the chance that participants with disease would experience signs and symptoms within the year of follow-up, *ψ *= 0.1; the variance, *σ*^2 ^= 1. The means of the ROC distributions for cases for Test 1 and Test 2 were 2.1 and 1.1, respectively, and the means for non-cases for Test 1 and Test 2 were 1.6 and 0.35. The correlation between test scores for cases was fixed at 0.1, as was the correlation for non-cases. All test scores above 2.5 on either test, or participants who had signs or symptoms had an infallible secondary test to determine disease status. For participants with scores below 2.5 on both tests, a less sensitive method was used to approximate disease status.

To understand how and why paired screening trial bias occurs, consider a single specificity value on the *true *and *observed *ROC curves shown in Figure 2. Choose the value of specificity where there is the greatest increase in *observed *sensitivity relative to *true *sensitivity, for Test 1. This occurs when specificity is 0.82. For a hypothetical study of 10,000 participants, and specificity of 0.82, the *observed *and *true *2 × 2 tables for Test 1 and Test 2 are shown in Figure 4.

Each one of the four tables uses a slightly different ROC cutpoint. For the *observed *table, Test 1 is positive if it exceeds 2.511; for the *true *table, Test 1 is positive if it exceeds 2.515. For the *observed *table, Test 2 is positive if it exceeds 1.269; for the *true *table, Test 2 is positive if it exceeds 1.265. The tables have different ROC cutpoints because they were chosen to have the same specificity, not the same cutpoint.

Also, the number of cases of disease observed in the study, 45, is much smaller than the true number of cases of disease in the population, 100. The *observed *number of cases of disease is smaller than the *true *number because not every participant undergoes the invasive, yet sensitive and specific secondary test, and thus some cases of disease are missed. The *observed *number of cases of disease is the denominator of the *observed *sensitivity. Because the denominator is smaller for *observed *sensitivity than for *true *sensitivity, the *observed *sensitivity is strongly inflated for both tests. When specificity is 0.82, the observed sensitivity of Test 1 is 0.72, with true sensitivity of 0.33. For Test 2, the observed sensitivity is 0.52, with true sensitivity of 0.43.

Yet if the bias only affected the denominator, the inflation in sensitivity would be the same for both tests. After all, the same number of observed cases is used as the denominator for both tests. The differential inflation for Test 1 compared to that for Test 2 must be due to the numerator of the observed sensitivity.

For Test 2, the numerator of the observed sensitivity is the number of study participants who are positive on Test 2, and who are observed to have disease in the study. For Test 2, the numerator for observed sensitivity, 23, is smaller than the true numerator, 43. The difference occurs because disease can only be observed if the invasive, yet sensitive and specific secondary test is used. Even though the participants have a score that exceeds the ROC cutpoint for Test 2, they do not all undergo the invasive, yet sensitive and specific secondary test. Thus, they do not yield observed cases of disease. By contrast, for Test 1, because the ROC cutpoint is higher than the threshold which leads to the invasive, yet sensitive and specific secondary test, every participant positive on Test 1 undergoes the secondary test, and is shown to have disease. For each test, there is a different proportion of participants who exceed the cutpoint, who truly have disease, and who proceed to secondary testing. This is the source of the differential bias that causes the curves to reverse order in Figure 2.

*Paired screening trial bias *also increases as the proportion of participants with disease who have signs and symptoms (*ψ*) decreases. If all the cases of the disease were observed during the trial, there would be no difference between *true *and *observed *disease status, and no bias. Yet, in every screening trial, some cases of diseases are not identified by either screening test, and never present with signs and symptoms. As the proportion of participants presenting with signs and symptoms (*ψ*) decreases, fewer cases of disease are discovered during the trial in the interval after screening, and the difference between *observed *and *true *disease status grows.

*Paired screening trial bias *increases with the increase in correlation between the results of the screening tests for participants with disease, *ρ*_*C*_. The bias in the *observed *ROC curves increases because as the two index tests become more highly correlated, the number of *observed *cases of disease becomes smaller relative to the number of *true *cases of disease. When the two index tests are highly correlated, they essentially produce the same information as to whether a participant has disease. When the index tests are independent, each test makes diagnoses on its own that the other test misses. Thus, when the tests are independent, and *ρ*_*C *_is 0, the number of *observed *cases is highest, relative to the number of *true *cases. The percentage of participants receiving the infallible secondary test increases as *ρ*_*C *_decreases. The bias lessens as the true disease status is ascertained for more participants.

In general, *paired screening trial bias *tends to strongly increase the sensitivity, while slightly decreasing the estimate of specificity. The increase in observed sensitivity compared to true sensitivity is expected with verification bias [9].

## Discussion

In this paper, we define a new type of bias that is a result of the interaction between a particular design for a paired screening trial, and the choice of a particular statistical test. Specifically, the bias occurs when the diagnostic accuracy of two continuous tests are compared using area under the ROC curve in a design with two limitations. First, different methods are used to ascertain disease status, depending on the results of the initial screening tests. Secondly, only some subjects undergo an invasive, yet sensitive and specific secondary test. Thus, some cases of disease are missed because the method used to ascertain disease status for those who test negative on both initial screening tests may not be 100% sensitive.

Both the statistical test and the trial design we considered were modeled closely after recently completed and published trials [1-3]. These trials compared the diagnostic accuracy of two modalities for breast cancer detection. Although authors have suggested the use of other statistical approaches to compare screening modalities [10,11], the area under the full ROC curve remains the most commonly used test for paired screening trials in major American journals [1-3].

Although we modeled our trial design on real trials, we made simplifying assumptions, which may not accurately reflect reality. We assumed that there was a method for determining disease status which was infallible. In reality, all methods of determining disease status may be fallible. In breast cancer, for example, diagnostic mammography, biopsy and follow-up all make errors. Too short a follow-up time may miss cases of disease. While longer follow-up time will reveal a larger fraction of occult disease, it may also reveal increasing numbers of cases of disease that developed after the initial screening period, thus confusing the results. We assumed that all cases of disease are harmful. In screening studies, cases of disease may resolve, or proceed so slowly as to be considered harmless.

We assumed that a test to determine disease status would be conducted any time a screening test result exceeded a given threshold. However, in cancer screening, because other factors may be taken into consideration when deciding a course of clinical action, there is a range of scores that may result in further testing.

We also made the simplifying assumption that the scores of the screening tests followed a bivariate normal distribution. In real paired cancer trials, the scores have a conditional probability structure driven by the fact that real observers miss cancers (and score a screening test as if no disease were present), and see cancer where there is none (and then score a non-cancerous finding as abnormal). The resulting distribution of scores is far from the bivariate normal distribution we assumed.

There is some theoretical justification that our results will still hold even if the data are non-normal. Hanley [12] points out that single test ROC analysis is robust to the violation of the normality assumption if there exists a monotonely increasing transformation of the test scores that yields a normally distributed result. Thus, the results described in the paper should hold whenever there is a transformation for screening Test 1, and another for screening Test 2 so that the transformed data has a bivariate normal distribution.

The previous literature on bias provides some hint of the plethora of possible designs and tests used for statistical analysis. Most previous statistical literature dealt with biases that occur for single, as opposed to paired, tests. A complete summary of biases is given in [4]. Extreme verification bias may occur when the diagnostic test is invasive or dangerous [5]. Verification bias has been studied in binary tests [13,14], and in ordinal tests [15,16]. Alonzo and Pepe [17] described using imputation and re-weighting to correct verification bias for single continuous tests. Alonzo [18] suggested corrections for verification bias in paired binary testing situations. We were unable to find published techniques to quantify or correct for *paired screening trial bias*.

Cancer screening trials in particular are susceptible to *paired screening trial bias*, because the secondary test is typically biopsy. Negative screening results cannot lead to biopsy because there is no visible lesion to be biopsied. Because biopsy is painful and invasive, it is infeasible and unethical to do a biopsy unless there are suspicious screening test results. Also, one can only biopsy what one can see: one cannot put a needle in an invisible lesion. Negative screening test results are verified, but typically by follow-up, which has lower sensitivity than biopsy.

Our research suggests that in many published paired screening trials, bias did not affect the scientific conclusion. For example, in Pisano *et al*., [2], digital and film mammography led to the recall of a very similar proportion of cases for the secondary test, diagnostic imaging. Thus, the trial design was more like Figure 3, in which bias occurs, but does not change the scientific conclusion, rather than Figure 2, in which bias occurs differentially, and changes the scientific conclusion.

### Epistemology

Why criticize a trial design, that though imperfect, cannot be improved, because of ethical constraints? It is our philosophy that it is preferable to understand all the causes of bias. With mathematical formulae for bias, we can defend trials that are fundamentally correct, and reserve doubt for those trials that may be subject to incorrect conclusions. In addition, models for bias are the necessary first step toward mathematical corrections for bias in sensitivity and specificity, and toward designing new clinical trial methodologies.

## Conclusion

Using a simplified paradigm, we have shown that *paired screening trial bias *has the potential to subvert the results of paired screening trials, especially when the fraction of the population recalled for secondary testing differs for each screening test. The bias is affected by the rate at which diseased participants experience signs and symptoms of disease, and the chance of recall for a sensitive secondary test. The bias is also influenced by the distributions of the scores for the cases and non-cases for each screening test, and by the correlation between the screening tests. Further research on this bias is needed, so that mathematical corrections for paired screening trial bias can be developed.

*Programs implemented in SAS and Mathematica to calculate the true and observed sensitivity, specificity, ROC curves, and areas under the curves are available by request from the authors*.

## Abbreviations

ROC: Receiver operating characteristic; AUC: Area under the receiver operating characteristic curve.

## Competing interests

Financial support for this study was provided by NCI K07CA88811, a grant from the National Cancer Institute to the Colorado School of Public Health, Deborah Glueck, Principal Investigator. The funding agreement ensured the authors' independence in designing the study, interpreting the data, writing, and publishing the report. The authors declare that they have no competing interests.

## Authors' contributions

DHG conceived of the idea and derived the mathematics. The first draft of the manuscript was a collaborative effort of DHG and MML. MML provided epidemiological expertise. CIO programmed the formulae and produced graphs. KEM provided advice about the mathematics. BMR and JTB aided in a thorough revision of the manuscript. JML and EDP provided clinical expertise and suggestions for how to relate the topic to medical studies. TAA assisted in the literature review, and in checking the math, and collaborated on the revision of the manuscript. All authors read and approved the final manuscript.

## Appendix

For those readers not familiar with ROC analysis, we give a short tutorial. For a complete discussion, see [[5];Chapter 4, pages 66–94] or [[19]; Chapter 4, pages 137–153]. The ROC curve is estimated by selecting a series of cutpoints. By convention, for each test, scores below the cutpoint are considered negative, and scores above the cutpoint are considered positive. The cross-classification of test results and disease status yields a set of two by two tables. Each table gives a paired estimate of sensitivity (the number of true positives correctly identified as positive by the test divided by the total number of cases) and specificity (the number of true negatives correctly identified as negative by the test divided by the total number of non-cases). The ROC curve for each test is graphed with sensitivity on the vertical axis, and 1 – specificity on the horizontal axis. The area under the curve (AUC) is a measure of the diagnostic accuracy of the test. A non-informative test follows the 45° line and has an AUC of 0.5. A perfect test follows the top and left boundaries of the ROC plot area, and has an AUC of 1.

## Pre-publication history

The pre-publication history for this paper can be accessed here:

http://www.biomedcentral.com/1471-2288/9/4/prepub
